# Positive Chemotactic Flasklike Colloidal Motors Propelled by Rotary F_o_F_1_-ATP Synthases

**DOI:** 10.34133/research.0566

**Published:** 2024-12-23

**Authors:** Yue Li, Yingjie Wu, Qiang He

**Affiliations:** School of Medicine and Health, Harbin Institute of Technology, Harbin 150001, China.

## Abstract

Living microorganisms can perform directed migration for foraging in response to a chemoattractant gradient. We report a biomimetic strategy that rotary F_o_F_1_-ATPase (adenosine triphosphatase)-propelled flasklike colloidal motors exhibit positive chemotaxis resembling the chemotactic behavior of bacteria. The streamlined flasklike colloidal particles are fabricated through polymerization, expansion, surface rupture, and re-polymerizing nanoemulsions composed of triblock copolymers and ribose. The as-synthesized particles enable the incorporation of thylakoid vesicles into the cavity, ensuring a geometric asymmetric nanoarchitecture. The chemical gradient in the neck channel across flasklike colloidal motors facilitates autonomous movement at a speed of 1.19 μm/s in a ΔpH value of 4. Computer simulations reveal the self-actuated flasklike colloidal motors driven by self-diffusiophoretic force. These flasklike colloidal motors display positive directional motion along an adenosine diphosphate (ADP) concentration gradient during adenosine triphosphate (ATP) synthesis. The positive chemotaxis is ascribed that the phosphorylation reaction occurring inside colloidal motors generates 2 distinct phoretic torques at the bottom and the opening owing to the diffusion of ADP, thereby a continuous reorientation motion. Such a biophysical strategy that nanosized rotary protein molecular motors propel the directional movement of a flasklike colloidal motor holds promise for designing new types of biomedical swimming nanobots.

## Introduction

Chemotactic migration in response to specific stimuli plays a crucial role in various bioprocesses [1,2]. Chemotaxis (migrating toward or away from chemical stimuli) enables microorganisms to navigate toward nutrients or evading toxic substances [3,4]. Drawing inspiration from biological systems, scientists have devised various enzyme-driven colloidal motors that can perform chemotactic movements by utilizing chemical-free energy from their surroundings [5–15]. These steerable colloidal motors hold considerable potential for practical applications [16,17], particularly in targeting therapy [18–21], drug delivery [22–27], and in situ imaging [28–30]. It has been proved that the directional motion of the assembled dual-enzyme-driven flasklike colloidal motors can be manipulated through the interaction of an externally applied glucose gradient and/or a self-generated gluconic acid gradient [31,32]. Regrettably, these motors are influenced by the ionic environment in their surroundings, just like other enzyme-powered colloidal motors, constraining their controllable mobility under physiological conditions.

On the other hand, rotary protein molecular motors such as F_o_F_1_-ATP (adenosine triphosphate) synthase, also known as F_o_F_1_-ATPase (adenosine triphosphatase), exhibit exceptional functionality under physiological conditions [33–36]. These protein motors harness the transmembrane proton gradient potential to facilitate the conversion of adenosine diphosphate (ADP) and inorganic phosphate (Pi) into bioenergy currency ATP [37–39]. Fascinatingly, the polymer microcapsules assembled with F_o_F_1_-ATPase motor proteins exhibit a biomimetic cellular migration by transforming naturally occurring energy in their surroundings into chemical energy ATP through metabolic reactions [40]. Furthermore, flasklike pentosan colloidal motors that incorporate rotary F_o_F_1_-ATPase protein motor-embedded hybrid liposomes can autonomously navigate within ionic-enriched physiological environments, and even in specific pH ranges [41]. These F_o_F_1_-ATPase-functionalized colloidal motors represent an excellent model for developing ATP delivery vehicles capable of active supply of ATP on demand [42]. Nevertheless, creating a supramolecular colloidal motor that integrates rotary protein motors, alongside the ability to control movement via ATP synthesis, is still largely uncharted territory and presents significant challenges.

Herein, we present a biohybrid flasklike colloidal motor powered by the collective catalytic action of a rotary biomolecular motor, exhibiting positive directional migration in ADP concentration gradient. The thylakoid vesicles containing F_o_F_1_-ATPases are infused into the cavity of flasklike colloidal particles to fabricate colloidal motors. The phosphorylation process of F_o_F_1_-ATPases within the cavity of flasklike colloidal motors could drive these motors' autonomously directional migration from the bottom toward the opening neck. The self-actuation speed depends on the diffusion of substances during the ATP synthesis process. Computer simulation reveals that the asymmetrical architecture of flasklike colloidal motors permitted ADP and Pi to diffuse into the cavity through the single opening, thereby ensuring the establishment of a localized chemical gradient across the colloidal motors for propelling. Moreover, the variations in diffusion rates between the bottom and the opening neck may generate a phoretic torque around these motors, resulting in the reorientation of their opening neck toward the source of the ADP gradient, thereby generating a positive chemotactic response.

## Results

### Construction and characterization

**Fig. 1.**
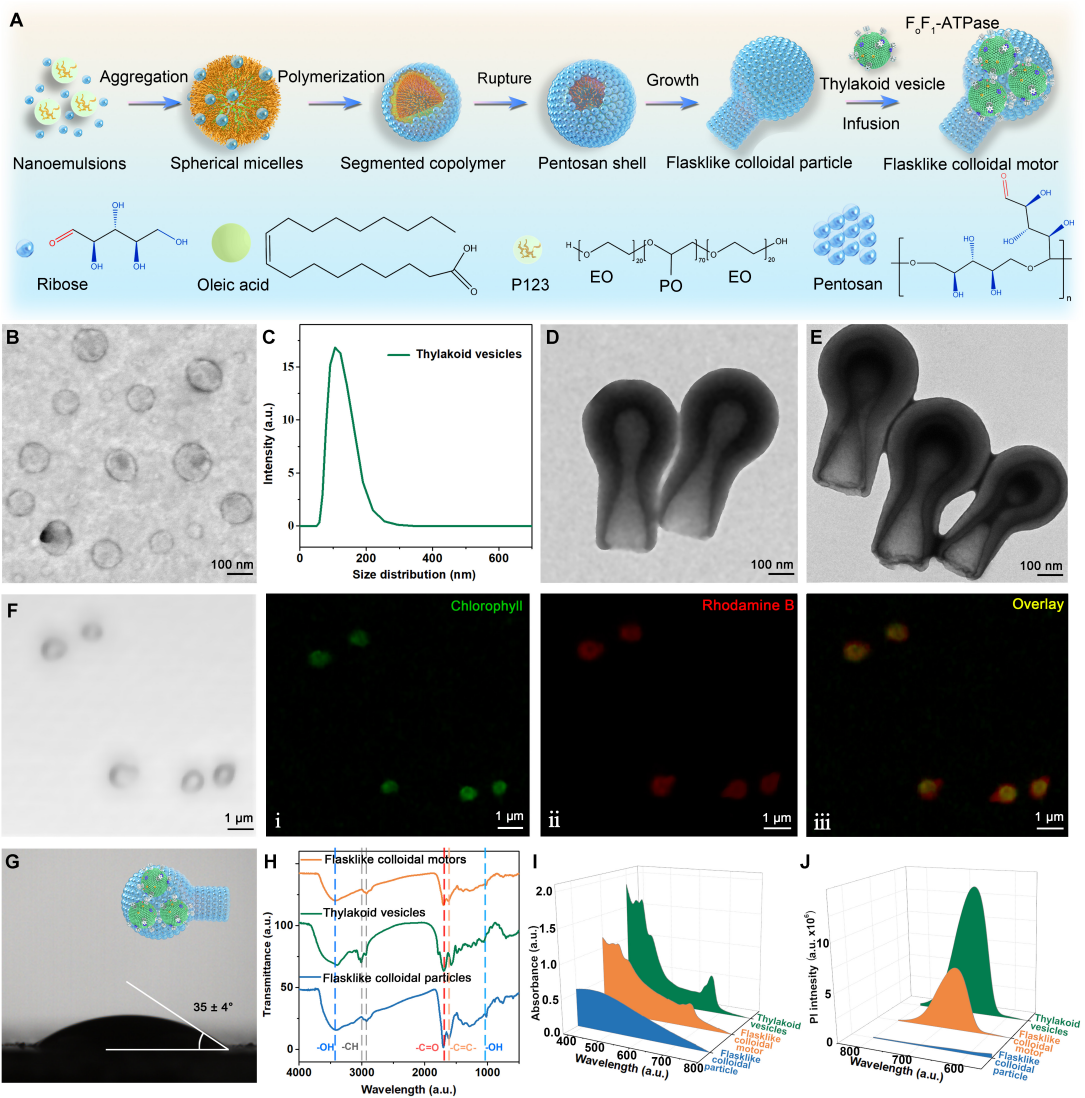
Schematic illustration of the hierarchical supramolecular assembly construction strategy and characterization of flasklike colloidal motors. (A) Schematic illustration of the hierarchical assembly of the flasklike colloidal motors. A schematic representation of the incorporation of thylakoid vesicles into the flasklike colloidal particle occurs under vacuum conditions, forming the flasklike colloidal motor. (B) TEM images of thylakoid vesicles. Scale bar, 100 nm. (C) DLS measurement of thylakoid vesicles. (D) TEM images of flasklike colloidal particles. Scale bar, 100 nm. (E) TEM images of flasklike colloidal motors. Scale bar, 100 nm. (F) CLSM images of flasklike colloidal motors. (i) The green fluorescence images (*λ*_ex_ = 488 nm) were obtained by exciting at 559 nm representing chlorophyll-containing thylakoid vesicles for green. (ii) The red fluorescence images (*λ*_ex_ = 559 nm) correspond to Rhodamine-stained flasklike colloidal particles. (iii) Overlapped fluorescence images of flasklike colloidal motor (chl = 2.3 mg/ml). (G) Water contact angle measurement of the flasklike colloidal motor monolayer (35 ± 4°). (H to J) FTIR spectra, UV-visible spectra, and fluorescence spectra of flasklike colloidal particles, thylakoid vesicles, and flasklike colloidal motors (*C*_chl_ = 2.3 mg/ml, absorption wavelength ranges from 400 to 800 nm; fluorescence wavelength ranges from 570 to 780 nm).

The construction of biohybrid submicrometer-sized flasklike colloidal motors using the supramolecular assembly strategy was depicted in Fig. 1A. Initially, P123 copolymers orchestrated the formation of spherical micelles in aqueous solutions at a low temperature of 30 °C. The core of the nanomicelle primarily consisted of polypropylene oxide (PO), which was enveloped by an external layer enriched with hydrated ethylene oxide (EO) blocks. With increasing temperature, the EO blocks exhibited heightened hydrophobic characteristics, leading to the enlargement of the micelles. Simultaneously, surface polymerization occurred within the nanoemulsion, impeding further aggregation. The increased hydrophobicity of the EO blocks in P123 promoted the expansion of the nanoemulsion, resulting in higher surface tension and eventually causing the rupture of the oligomerized shell. At low concentrations, hydrophobic pentosan intermediates infiltrated the core, displacing the surfactant and triggering polymerization on the walls of the cavity. The displaced surfactant establishes a new interface at the rupture site, serving as a soft template for the subsequent polymerization of intermediates at this interface, ultimately leading to the formation of flasklike colloidal particles, as evidenced in previous investigations [43]. The thylakoid vesicles were synthesized as previously documented methodologies (Fig. S1) [44,45]. Briefly, the fresh spinach leaves were ground to create a homogenate of mesophyll cells, followed by the isolation of chloroplasts through gradient density centrifugation. Subsequently, the chloroplasts were mixed with a hypotonic solution to release the thylakoids as detailed in previous studies [46]. The intact thylakoids were homogenized with a glass homogenizer to produce thylakoid membrane fragments. These fragments were transformed into thylakoid vesicles by mechanically extruding them utilizing a porous polycarbonate membrane with a 100 nm diameter. Finally, the F_o_F_1_-ATPase-contained thylakoid vesicles were obtained. The as-assembled F_o_F_1_-ATPase-contained thylakoid vesicles were infused into the hollow cavity of flasklike colloidal particles to obtain the flasklike colloidal motors. As shown in Fig. S2, the chloroplasts and thylakoids exhibited a crescent-shaped hemispherical morphology, with dimensions ranging from 5 to 10 μm. The homogenization procedure enabled the transformation of intact thylakoids into thylakoid membrane fragments. Also, the scanning electron microscopy (SEM) images of the chloroplast and thylakoid demonstrated a stacked arrangement of grana and stromal architecture, with a 7 μm diameter (Fig. S3). Transmission electron microscopy (TEM) images reveal that thylakoid vesicles present a small unilamellar vesicle structure (Fig. 1B), which exhibits a mean diameter of 100 ± 40 nm, as confirmed by dynamic light scattering (DLS) data in Fig. 1C. The TEM image in Fig. 1D and the SEM image (Fig. S4) demonstrate that the flasklike colloidal motors possess a flask-like architecture featuring an opening neck. The flasklike colloidal particles exhibit a body length of 850 ± 45 nm, an external neck diameter of 420 ± 40 nm, and a spherical bottom diameter of 400 ± 50 nm. Moreover, the TEM image in Fig. 1E demonstrates that the dimensions and architectural features of the as-prepared flasklike colloidal motor are consistent with the flasklike colloidal particle. More importantly, the dark regions observed inside the cavity of the flasklike colloidal motor suggest the effective encapsulation of thylakoid vesicles.

Confocal laser scanning microscopy (CLSM) images revealed green fluorescence signals localized within the cavities of the flasklike colloidal motors, which is derived from the natural chlorophyll present in thylakoid membranes, upon excitation at a wavelength of 488 nm. Conversely, the red signals observed are attributed to the rhodamine staining of the flasklike colloidal particles when excited at 536 nm, as depicted in Fig. 1F. To verify the incorporation of thylakoid vesicles in the cavity of the flasklike colloidal motors rather than merely sticking to the surface, the contact angle of flasklike colloidal particles and colloidal motors were examined. Note that the contact angle of flasklike colloidal particles (Fig. S5) and flasklike colloidal motors (Fig. 1G) were 30 ± 2° and 35 ± 4°, respectively, indicating that the as-prepared motors retained their original surface wettability not affected by the infusion of thylakoid vesicles. The thylakoid vesicles, originating from the native thylakoid protein-enriched phospholipid membrane, were characterized by a Fourier infrared spectrometer (FTIR). Specifically, the peak at 1,700 cm^−1^ was associated with -C=O stretching, the peak at 1,640 cm^−1^ corresponded to -C=C- stretching, and the peak at 1,096 cm^−1^ was assigned to the stretching vibration mode of -OH. These spectral features are also observed in both flasklike colloidal particles and colloidal motors. Furthermore, the peak at 2,830 cm^−1^, attributed to hydroxyl -CH stretching, aligns with the -CH stretching peak in the flasklike colloidal particles, also evident in the colloidal motors. In other words, the component of F_o_F_1_-ATPase-embedded thylakoid vesicles also persisted within the flasklike colloidal motors (Fig. 1H). Furthermore, the ultraviolet (UV)-visible absorption spectra demonstrate consistent absorption bands at 435 and 676 nm for both thylakoid vesicles and flasklike colloidal motors (Fig. 1I). The fluorescence spectra further indicate the presence of the characteristic chlorophyll emission peak at 687 nm (λ_ex_ = 488 nm) in both flasklike colloidal motors and thylakoid vesicles, as shown in Fig. 1J. This observation confirms the presence of pigments (e.g., chlorophyll a, chlorophyll b, carotene, and lutein) [47] derived from the thylakoids. Taken together, all these findings confirmed the effective integration of F_o_F_1_-ATPase-embedded thylakoid vesicles into the cavities of flasklike colloidal motors.

### Self-propulsion motion

**Fig. 2.**
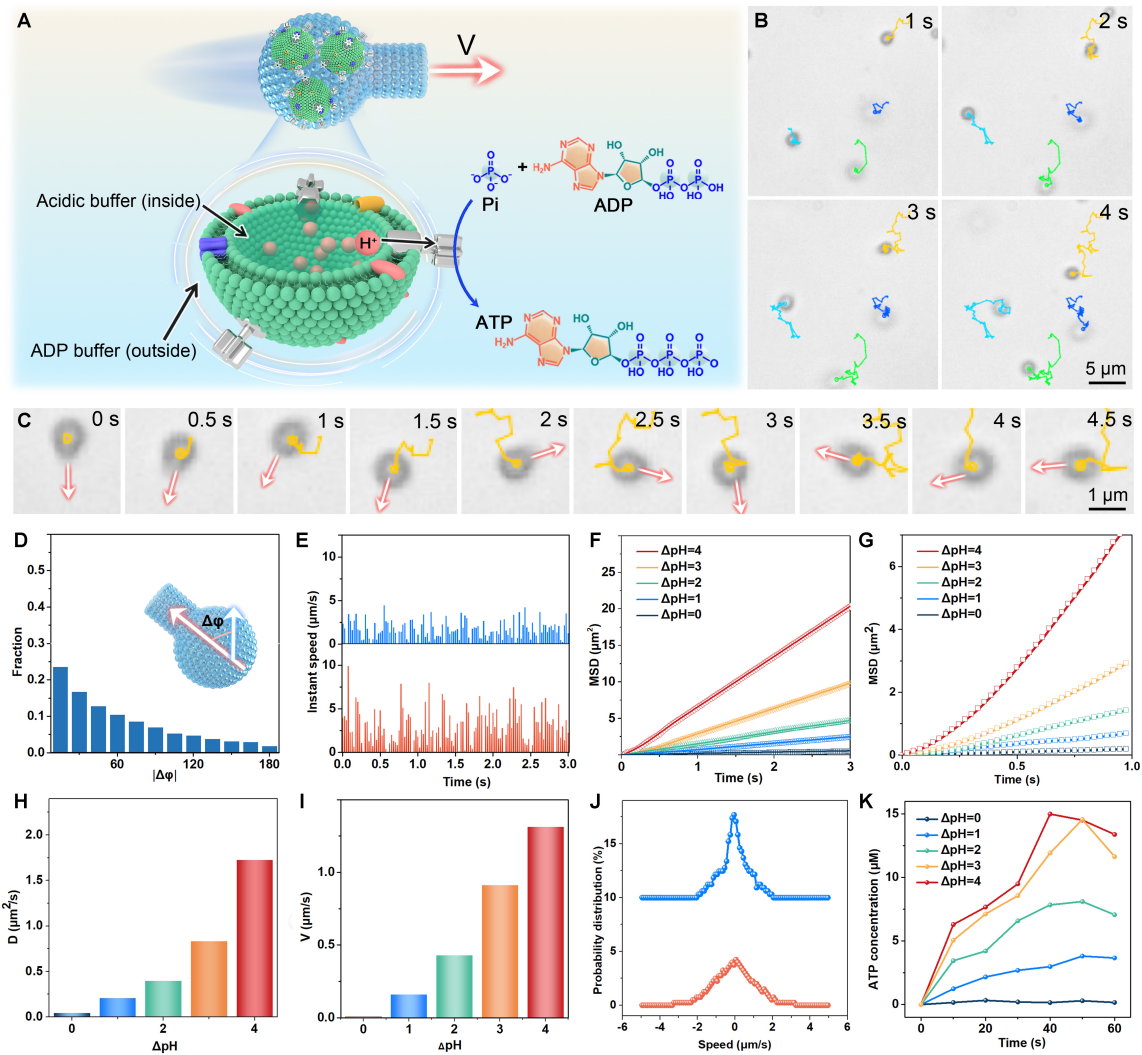
Autonomous motion behavior of flasklike colloidal motors under variable ΔpH values. (A) Schematic depiction of the phosphorylation process occurring on the surface of thylakoid vesicles inside of flasklike colloidal motors (swimming from the bottom to the opening neck during the ATP synthesis process). (B) Time-lapse images of the self-propelled motion of the flasklike colloidal motors in the ADP buffer for 4 s. Scale bar, 5 μm. (C) Enlarged screenshot of the trajectories of flasklike colloidal motors during autonomous motion. Scale bar, 1 μm. (D) Distribution of the orientational angle Δφ of the flasklike colloidal motors in the ADP buffer (ΔpH = 4) for 5 s. (E) Instant speed of self-actuated flasklike colloidal motors (driven by F_o_F_1_-ATPases via phosphorylation in a condition of ΔpH = 4, Δ*t* = 3 s) and Brownian motion (ΔpH = 0, Δ*t* = 3 s) of flasklike colloidal motors. (F) MSD curves of the flasklike colloidal motors at different ΔpH values in time interval analysis (Δ*t* = 3 s, number = 25). (G) MSD of the flasklike colloidal motors at different ΔpH values in time interval analysis (Δ*t* = 1 s, number = 25). (H) Self-propelled speed (*V*) of flasklike colloidal motors at different pH gradients (number = 25). (H) Corresponding effective diffusion coefficient *D*_eff_ (Δ*t* = 3 s) of flasklike colloidal motors at different pH value conditions. (I) Self-propelled speed (*V*) of flasklike colloidal motors at different pH gradients. (J) Speed probability density distribution on the *x* axis of flasklike colloidal motors (ΔpH = 4) and Brownian motion of flasklike colloidal motors (ΔpH = 0). (K) Average rate of ATP production of flasklike colloidal motors in surroundings in 60 s (*C*_chl_ = 2.3 mg/ml).

A strategy based on acid-base transactions was employed to generate transmembrane proton gradients that drive the rotation of F_o_F_1_-ATPases within the cavity of the flasklike colloidal motors, as scheme illustration in Fig. 2A. Before the incorporation of thylakoid vesicles, the as-assembled thylakoid vesicles were incubated in acidic solutions with variable pH values from 4.8 to 7.0 (Table S1), leading to a decreased pH level of the internal liposomal phase (pH_in_) accordingly. Then, the as-assembled flasklike colloidal motors were mixed with a higher-pH buffer (ranging from pH 7.0 to 8.8), representing the internal liposomal phase’s acidity level (pH_out_). In this case, the transmembrane proton potential of the thylakoid vesicles was established. The time-lapse images (Fig. 2B and C; see also Movie S1) show that these flasklike colloidal motors can move along a direction from the bottom to the single opening once generating a pH difference (ΔpH = 4) between the interior and exterior pH values. The directional motion was further examined by monitoring the flasklike colloidal motors, specifically analyzing the angle between the directions of subsequent movements and orientation (Δϕ), which were defined as schematically illustrated in the inset of Fig. 2D. Subsequently, the angle Δφ between the polarities of the motors was measured over 14 s (Fig. 2D). It is further apparent that the flask-shaped colloidal motors tended to move from the rounded bottom toward the opening neck.

These flasklike colloidal motors demonstrate a remarkable capacity for self-actuated motion exposure to an ion-enriched environment. Specifically, the instant velocity (*V*) of the flasklike colloidal motors in ADP buffer solution, with pH differences of ΔpH = 0 and ΔpH = 4, exhibited significant variation, which may be ascribed to the chemical diffusion processes across the flasklike colloidal motor during the ATP synthesis process (Fig. 2E). The maximum instantaneous velocity of the flasklike colloidal motor reached 9.6 μm/s (corresponding trajectory in Fig. 2B). To explore the propulsion efficiency of flasklike colloidal motors under different reaction conditions, we charted the mean square displacement (MSD) [48] over time intervals according to the following equation (Eq. 1):

$$MSD=\left(4D+V^{2}\tau_{R}\right)\;\Delta t$$ (1)

where D denotes the translational diffusion coefficient, Δt means the time interval, and $\tau_{R}$ is the rotational time. The MSD curves exhibited a linear relationship at ΔpH = 0, demonstrating the typical behavior of Brownian motion (see Movie S2). Conversely, the MSD curves demonstrated a parabolic shape at ΔpH = 1, 2, 3, and 4, with the slopes increasing as the pH value of the ADP buffer solution increased (Fig. 2F). Particularly, the MSD curve exhibits a quadratic relationship at shorter time scales, before evolving into a linear relationship at extended time scales as illustrated in Fig. 2G. For assessment of the self-propulsion of flasklike colloidal motors, the diffusion coefficient [49] (i.e., effective diffusion coefficient, *D*_eff_) was derived using the following equation (Eq. 2).

$$D_{\text{eff}}=D+1/4V^{2}\tau_{R}$$ (2)

As shown in Fig. 2H, $D_{\mathrm{eff}}$ was increased as the ΔpH value of the ADP buffer solution increased, whose diffusion coefficients were 0 μm/s at ΔpH = 0, 0.18 μm/s at ΔpH = 1, 0.45 μm/s at ΔpH = 2, and 0.86 μm/s at ΔpH = 3, and the self-propelling speed of flasklike colloidal motors reached a maximum of 1.19 μm/s at ΔpH = 4 (Fig. 2I). Besides, the probability of *x*-directional velocity distribution [50] of Brownian motion in 1-s interval displays a prominent peak Gaussian distribution, whereas the self-actuated flasklike colloidal motors exhibit a more gradual peak Gaussian distribution, further indicating the self-propulsion of flasklike colloidal motors (Fig. 2J).

Subsequently, the ATP synthesis by F_o_F_1_-ATPases on the flasklike colloidal motors was examined. The concentration of as-produced ATP was quantified using liquid chromatography. The ATP concentration gradually increased in 50 s under ΔpH values = 1, 2, 3, and 4, respectively. Specifically, the final concentration of ATP in the solution reached 13.4 μM at ΔpH = 4 after 60 s (Fig. 2K). Also, the rate of ATP production was positively related to the self-propelling speed of flasklike colloidal motors. Given the presence of phosphorylation cascade reactions, we hypothesize that the self-propelling motion of the flasklike colloidal motor is driven by F_o_F_1_-ATPases, during the ATP synthesis process.

### Self-propulsion mechanism

**Fig. 3.**
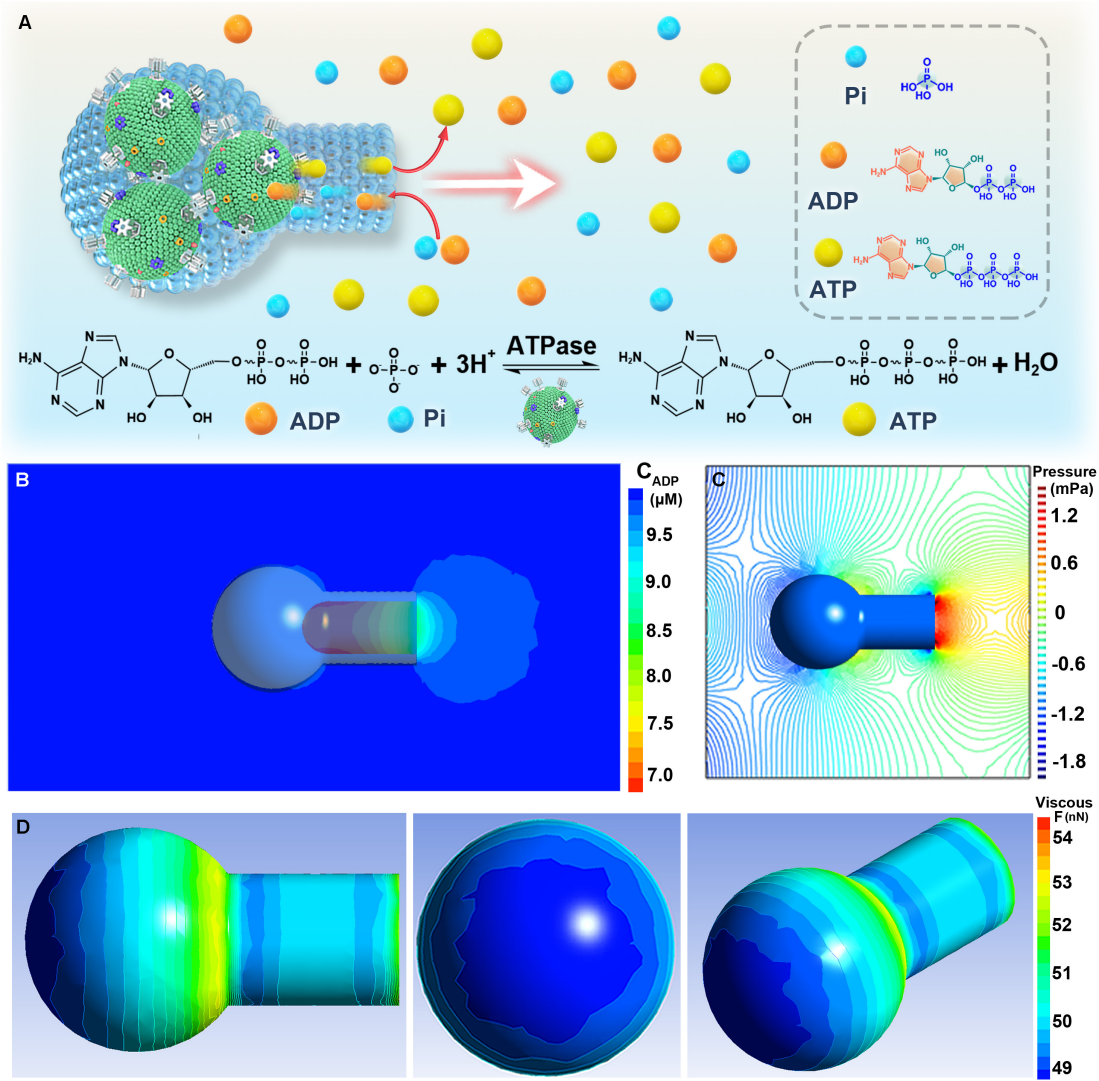
Analysis of the self-propulsion mechanism of the flasklike colloidal motors. (A) Schematic illustration of the motion of the flasklike colloidal motors. In the presence of an ADP buffer, a transmembrane proton gradient was generated to drive multiple F_o_F_1_-ATPase rotation and transform ADP into ATP via phosphorylation. (B) Computer simulation on the ADP concentration distribution and the corresponding local flow fields caused by the phosphorylation reaction of flasklike colloidal motors. (C) Pressure distribution around the flasklike colloidal motors induced by ADP diffusion. (D) Viscous force distribution during the flasklike colloidal motor swimming from the bottom toward the opening neck and 3-dimensional rebuild viscous force distribution of surface viscous force on the flasklike colloidal motor, where the arrows refer to the flow velocity.

In our previous investigation, the rotation of F_o_F_1_-ATPase played a negligible role in the propulsion of the flasklike colloidal motors [40]. Given this, the self-propelling force of flasklike colloidal motors could be predominantly attributed to phosphorylation-induced chemical diffusion. More importantly, the hollow flasklike architecture of flasklike colloidal motors allows solute diffusion through the narrow inner neck (∼80 ± 20 nm as shown in Fig. 1D). During the phosphorylation reaction, ADP and Pi migrate from the single opening into the cavity and ATP leaks out from the neck of flasklike colloidal motors (Fig. 3A). The diffusion contributes to the self-diffusiophoretic force acting on the flasklike colloidal motors. To evaluate this hypothesis, we conducted a computer simulation that delineated the concentration of ADP in the vicinity of the flasklike colloidal motors, employing a finite element multiphysics coupling methodology. A concentration gradient of ADP was generated along the opening to the narrow inner neck of flasklike colloidal motors due to the internal proton consumption by the F_o_F_1_-ATPases, as indicated in Fig. 3B. Consequently, the ADP concentration gradient facilitated local flow, resulting in a difference in fluid pressure across the flasklike colloidal motors (Fig. 3C). To better assess the impact of fluid pressure, the distribution of viscous force around the flasklike colloidal motors was calculated as follows (Eq. 3):

$$F_{v}=0.5\;\rho \;A\;v^{\wedge }2C_{d}$$ (3)

where $F_{v}$ represents the viscous force, *ρ* denotes the density of the fluid, A means the cross-sectional area of the object perpendicular to the flow direction, v is the velocity of the object relative to the fluid, and $C_{d}$ signifies the drag coefficient, which is influenced by architecture and surface properties of the object. The simulation results proved that the viscous force at the neck joint and opening is much stronger than the round bottom (Fig. 3D), which consists of the fluid pressure distribution (Fig. 3C). Taken together, the concentration gradient of the reactants ADP and PO_4_^3−^ generated by the coordinated rotation of F_o_F_1_-ATPase biomolecular motors produces a self-diffusiophoretic force that facilitates the autonomous motion of the flasklike colloidal motor.

### Directional motion analysis

**Fig. 4.**
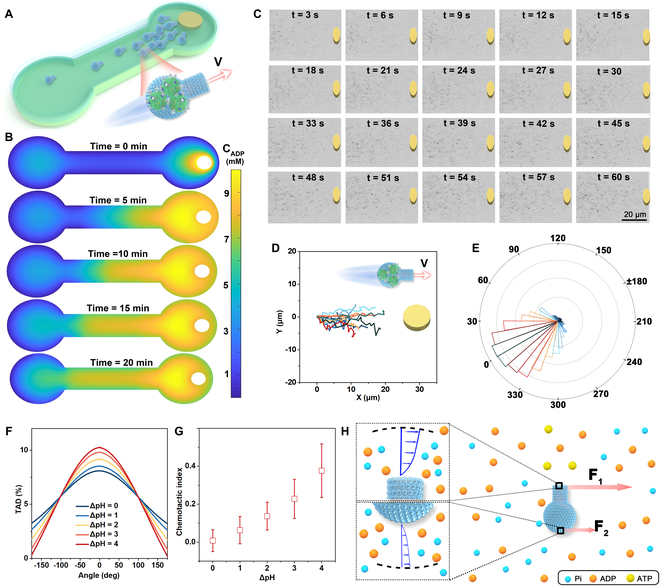
Directional migration of the flasklike colloidal motors under different ΔpH values. (A) Schematic diagram of the chemotactic migration of flasklike colloidal motors along the ADP gradient via phosphorylation. (B) Computational simulation of the diffusion profile of ADP within a microfluidic channel at different time intervals (0 to 25 min). (C) Time-lapse images of the chemotactic behavior of the flasklike colloidal motor. Scale bar, 5 μm (1 min). (D) Corresponding normalized trajectories of 10 flasklike colloidal motors in 5 s. (E) Direction distribution of flasklike colloidal motors in an ADP gradient at different pH value conditions (*n* = 2,000). (F) TAD of flasklike colloidal motors at different pH gradient sources in a time interval of 3 s (*n* = 6,000). (G) CI of the flasklike colloidal motors at different pH gradient conditions (*n* = 6,000). (H) Scheme illustration of the chemotactic mechanism of the flasklike colloidal motors under an ADP gradient.

To assess the chemotactic behavior of these hydrophilic flasklike colloidal motors, a cylindrical agarose gel pre-soaked in an ADP buffer solution overnight was positioned on the right side of the water-filled microfluidic channel to generate an ADP gradient (Fig. 4A). Following the establishment of the ADP gradient, the flasklike colloidal motors were introduced into the central region of the petri dish. Simulation results indicated that ADP gradually diffused from the agarose gel in the form of concentric circles along the microchannel, resulting in a gradual decrease in ADP concentration with increasing distance from the agarose gel in the microfluidic channel over 20 min, as shown in Fig. 4B. Time-lapse images of the flasklike colloidal motors in response to the ADP gradient (Movie S3) revealed their sensibility to the local ADP gradient, subsequently aligning and migrating toward the ADP source, as depicted in Fig. 4C. Also, the chemotactic migration of the flasklike colloidal particles within the ADP buffer gradient was investigated (Fig. S6). As the duration of chemotaxis increased, the ADP gradient facilitated a migratory flow. Upon introduction into the chemotactic channel, the flasklike colloidal particles exhibited movement away from the agarose gel containing the ADP buffer, attributed to the diffusiophoresis of the solute. Notably, only the flasklike colloidal motors within the ADP buffer gradient demonstrated positive chemotactic motion along the gradient, as evidenced by the normalized trajectories in Fig. 4D. The angle distribution of the chemotactic movement of the flasklike colloidal motors ranged within ± 35° along the direction of the ADP gradient (Fig. 4E), supporting the notion that these motors could autonomously perceive a concentration gradient and move toward the ADP source. To further elucidate the biased movement toward the ADP source, the turning angle distribution (TAD) [32] of the flasklike colloidal motors was introduced by measuring the displacement distribution. The TAD exhibited a uniform directional distribution across all angles at ΔpH = 0. However, in the presence of the ADP buffer, significant peaks were observed at 0°, with the TAD at this angle increasing in correlation with the rising pH values of the ADP buffer (Fig. 4F). These findings indicate that the ATP synthesis process drives the directional motion of the flasklike colloidal motors, with the self-propelling force being contingent upon the intensity of the F_o_F_1_-ATPase cascade reaction. Meanwhile, as the duration of chemotaxis increases, the flasklike colloidal motor exhibits an enhanced accumulation in the fixed area near the substrate source (Figs. S7 and S8). As the flasklike colloidal motors moved toward the ADP source, their drift velocity increased from 0 to 1.03 μm/s, indicating that the flasklike colloidal motors demonstrated heightened chemoattractant by the substrate ADP source (Fig. S9). Additionally, we evaluated the migration persistence through the chemotactic index (CI), which quantifies the ratio of total displacement to the length of the channel. As shown in Fig. 4G, the CI of flasklike colloidal motors exhibited a positive correlation with the intensity of the phosphorylation cascade reaction.

To explain the self-reorientation behavior, a hypothesis is proposed by considering chemical diffusion processes, as illustrated in Fig. 4H. Infusing thylakoid vesicles within the flasklike colloidal motors allows them to generate self-propelled force through substrate (ADP and Pi) consumption and production (ATP) diffusion. Consequently, the ADP concentration at the opening is consistently lower than that at the round-bottom internal of the flasklike colloidal motor. This discrepancy in chemical concentration gradients (∇c_ADP_) results in a surface slip velocity (V_slip_) given by Eq. 4

$$V_{\text{slip}}=k_{B}T\;c/\eta$$ (4)

where $k_{B}$ denotes the Boltzmann constant, T represents the temperature, and η signifies viscosity. The heterogeneous distribution of ADP results in inconsistency with surface slip flow [51,52] $\left(V\propto \text{∇}c,V_{\text{slip}}-{V\prime }_{\text{slip}}\right)$. This variation in the surface slip flow provides the disparity of self-diffusiophoretic forces $\left(F\propto V_{\text{slip}},\;F_{1}>F_{2}\right)$, which thus prompts the reoriented motion. Accordingly, the asymmetrical diffusiophoretic forces generated by the nonuniform concentration gradient induce a torque, facilitating the alignment of flasklike colloidal motors in parallel with the ADP concentration gradient. This result indicates that the motors possess a robust capability during ATP synthesis and can autonomously respond to the chemical signals, thereby enabling chemotactic movement.

## Conclusion

We have successfully demonstrated the positive directional motion and ATP synthesis capabilities of rotary F_o_F_1_-ATPase-propelled flasklike colloidal motors. The distinctive submicrometer-scale and streamlined flasklike nanoarchitecture of colloidal motors allow the loading of rotary F_o_F_1_-ATPase protein motor-bound thylakoid vesicles. Consequently, ADP, Pi, and ATP diffusion are restricted to the openings and narrow neck channels of flasklike colloidal motors. An internal transmembrane proton gradient generates force to drive F_o_F_1_-ATPases to synthesize ATP in the cavity. As a result, ADP and Pi molecules are utilized as reactants in the ATP synthesis process. Due to the inherent ion insensitivity of natural F_o_F_1_-ATPase, flasklike colloidal motors can autonomously migrate effectively in ion-rich environments during phosphorylation. In conjunction with computer simulations, the self-propelling mechanism of these colloidal motors is identified as self-diffusiophoresis, which is induced by the diffusion of ADP. More interestingly, these flask-like colloidal motors can autonomously perceive ADP concentration gradients and exhibit positive chemotaxis while synthesizing ATP. Owing to their submicrometer dimensions, biocompatibility with fuels (ADP), and substantial loading capacity, these flask-shaped colloidal motors, which exhibit pronounced chemotactic properties, have the potential to function as active agents for targeted delivery. Such untethered, steerable, and biomolecular rotary molecular motor-powered flasklike colloidal motors enable the development of a therapeutic strategy by targeting supplying bioenergy ATP for biomedical applications in the future.

## Methods

### Preparation of the thylakoid vesicles

Chloroplasts were separated from fresh spinach using a method similar to that reported previously. Briefly, 500 g of fresh spinach was ground with quartz sand in a mortar and added to a chloroplast buffer containing 0.4 M sucrose, 0.01 M KCl, 0.01 M Na_2_HPO_4_, 0.03 M KH_2_PO_4_, and 2 mM vitamin C in 40 ml. The homogenate was collected in a centrifuge tube and filtered through 6 layers of cotton gauze. The supernatant was collected after centrifuging the filtrate at 3,000*g* for 2 min. Gradient density centrifugation (20%, 40%, and 60% sucrose solution) was used to separate intact chloroplasts during the second centrifuge filtrate. The intact chloroplasts were washed 3 times with 10 ml of buffer, and a green suspension was obtained. The recycled chloroplast pellet was suspended in a solution containing 10 mM Hepes-KOH (pH 7.8) and 5 mM MgCl_2_ and allowed to stand for 15 min in the dark on ice to lyse the chloroplasts. Intact thylakoids were collected by centrifuging at 3,000*g* for 3 min and then resuspended in a solution with 50 mM Hepes-KOH (pH 7.5), 100 mM sorbitol, and 10 mM MgCl_2_. The stacked thylakoids were homogenized in a glass homogenizer for 5 min in an ice bath. Then, thylakoid fragments were mechanically extruded via a porous polycarbonate membrane (100 nm) to fabricate thylakoid vesicles. Finally, the clear light green solution was stored in the dark at 4 °C.

### Preparation of the flasklike colloidal motors

The flasklike colloidal motors were fabricated using a previously established method. In brief, a clear solution was formed by slowly stirring 0.0365 g of sodium oleate (SO) and 0.0435 g of poly (ethylene glycol)-block-poly (propylene glycol)-block-poly-(ethylene glycol) (EO_20_-PO_70_-EO_20_, P123) in 20 ml of ultrapure water. Subsequently, 40 ml of a solution containing 3 g of ribose was added and the resulting mixture was stirred for 30 min before being transferred to a 100-ml autoclave and placed in an oven at 160 °C for 10 h. The resulting products were collected, washed by centrifugation, and then dried and stored as flasklike colloidal particles. To prepare the flasklike colloidal motors, 1 mg of the particles was added to a solution containing 20 mg of thylakoid vesicles in 2 ml of ADP buffer solution. This solution was placed in a vacuum chamber at a pressure of −0.1 bar for 30 min and then ultrasonicated for 1 h at 4 °C. The resulting motors were centrifuged and washed 3 times with ADP buffer solution and finally stored in the same solution at 4 °C.

### Evaluation on ATP production

ATP production was quantified using high-performance liquid chromatography (HPLC). The HPLC system employed for this analysis was the UltiMate E3000, manufactured in the Netherlands, which included a degasser, a quaternary pump with a manual injector, a lamp, and a UV detector. The mobile phase consisted of 0.1 M ammonium dihydrogen phosphate (NH_4_H_2_PO_4_) adjusted to a pH of 6.0, supplemented with 1% methanol. A reversed-phase fully porous silica (C^18^) column was utilized, characterized by an internal diameter of 4.6 mm, a length of 250 mm, a particle size of 5 μm, and a pore size of 300 Å. The UV detection wavelength was conducted at a wavelength of 254 nm, with a sample loop volume of 20 μl and a flow rate of 1 ml/min. Each sample was measured over 10 min. Before measurement, dithiothreitol (DTT) was introduced to the proteoliposomes at a final concentration of 50 mM, allowing for enzyme reduction to occur over 0.5 h. Additionally, the flasklike colloidal motors were incorporated into 1 ml of the buffer solution (pH 8.0, 10 mM tricine-NaOH, 20 μM ADP, 5 mM NaH_2_PO_4_, 2.5 mM MgCl_2_, and 30 mM NaCl). Sampling every 10 min monitoring of composition changes.

### Optical video recording

To clearly observe the chemotaxis of flasklike colloidal motors, an Olympus IX71 inverted microscope was employed. The petri dish was filled with acidic buffer solution to a height of 1 mm, and the agarose gel (1 mm diameter and height) containing acidic buffer was placed on the right side of the dish. After 10 min, 100 μl of water containing flasklike colloidal motors (1 mg ml^−1^) was injected on the opposite side of the agarose gel. The ADP buffer agarose gel was placed on the left or upper side. The flasklike colloidal motors were replaced with flasklike colloidal particles in these experiments.

### Numerical simulations

A computational simulation of the procedure is conducted utilizing the COMSOL finite element analysis software. The utilization of the chemical reaction engineering and dilute chemical species transport modules is employed. F_o_F_1_-ATPase was presumed to asymmetry distribute within the flasklike colloidal motor with a numerical value of 23. When the nanobot moved in ADP solution through phosphorylation, external chemical reactions ADP + Pi→ATP occurred on the surface of the nanobot. The diffusion coefficient (*D*) of ADP and Pi was set to be 0.145 × 10^−9^ and 0.327 × 10^−9^ m^2^ s^−1^, respectively. In order to optimize the computational efficiency and improve the convergence of results, the surface chemical reaction engineering module has been substituted with the convection–diffusion equation with specific boundary conditions to determine the concentration field *c* of the chemical species as follows:

$$\nabla J_{i}=u\nabla c_{i}-D_{i}\nabla^{2}c_{i}$$ (5)

where $J_{i}$ refers to the ionic flux, $c_{i}$ represents ionic concentration for species i, u is the flow velocity, and D is the ionic diffusion coefficient. The chemical reaction ADP + Pi→ATP took place in the cavity of the flasklike colloidal motor, and the boundary fluxes at the catalytic surfaces as follows:

$$n\cdot \left(-D_{i}\nabla c_{i}\right)=R_{\mathrm{ads},i}\;n$$ (6)

Under the chemical gradient of ADP, a chemiosmotic surface slip was initiated on the surface of flasklike colloidal motor. Upon securing the flasklike colloidal motor within the model, we established the surface of the motor as a slip boundary condition. Furthermore, we integrated chemiosmotic flow with a slip velocity, as follows:

$$u=\left(I-\mathrm{nn}\right)(b_{N}\cdot \;\sum \left(\nabla \text{cions}\right)$$ (7)

where b_N__ionic is the surface mobility of the flasklike colloidal motor, caused by concentrations of ionic species. The quantity $\left(I-\mathrm{nn}\right)$ defines the concentration gradients of ionic species, where I denotes the second-order unit tensor. The fluid dynamics surrounding the flask-shaped colloidal motor were analyzed using the Stokes equation as follows:

∇p=η∇2u,∇·u=0 (8)

where p is the pressure and η is the dynamic viscosity of water.

## Data Availability

All data are available from the corresponding author upon request.
