# Supplementary material for: Positive Chemotactic Flasklike Colloidal Motors Propelled by Rotary FoF1-ATP Synthases
Source: Research (Wash D C). 2024 Dec 23;7:0566. doi: 10.34133/research.0566 (PMC11665525; doi:10.34133/research.0566)
Supplement: Supplementary 1 — Figs. S1 to S9 Table S1 Movies S1 to S3 [file research.0566.f1.zip › Supplementary Figures.pdf]

Research A SCIENCE PARTNER JOURNAL
SPJ SCIENCE PARTNER JOURNALS

Supplementary Figures

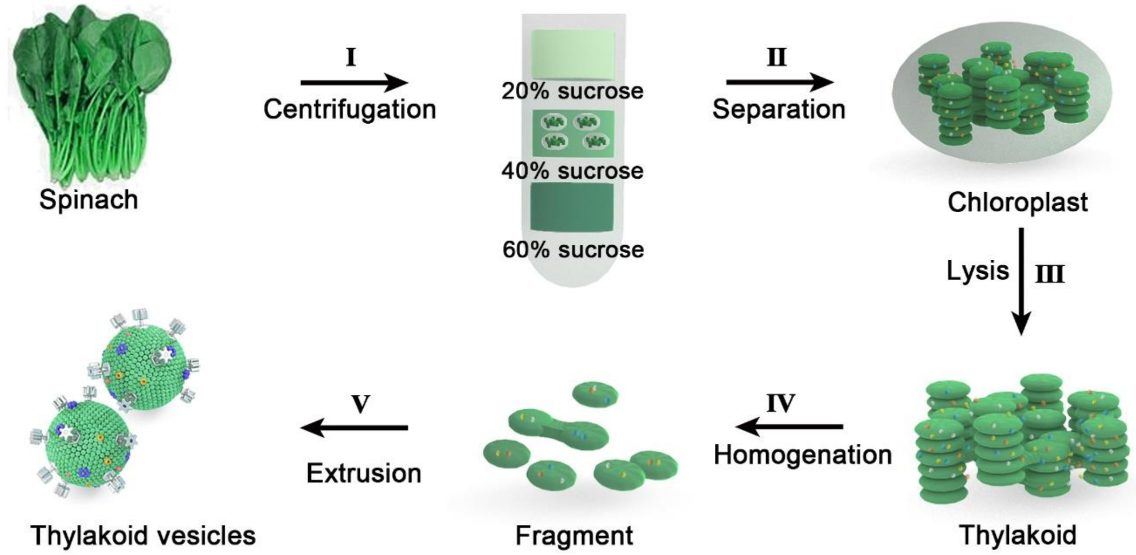

Figure S1. Schematic illustration of the hierarchical supramolecular assembly and plant-derived thylakoid vesicles. (I) Spinach leaves were first ground, and then the mesophyll cell homogenate was transferred in a centrifuge tube; The homogenate was centrifuged to recover the sediments containing chloroplasts, nucleus, and other impurities; (II) Chloroplasts were separated through differential centrifugal sedimentation and further purified by sucrose gradient density centrifugation; (III) The recycled chloroplasts were destroyed by hypotonic lysis and the released thylakoids were collected; (IV) The thylakoid suspension was homogenized by using a glass tissue homogenizer and the supernatant containing thylakoid membrane fragments was recovered; (V) The thylakoid membrane fragments containing rotary F_oF_1-ATPase protein motors were co-extruded through the porous membrane (100 nm) to assemble the thylakoid membrane vesicles.

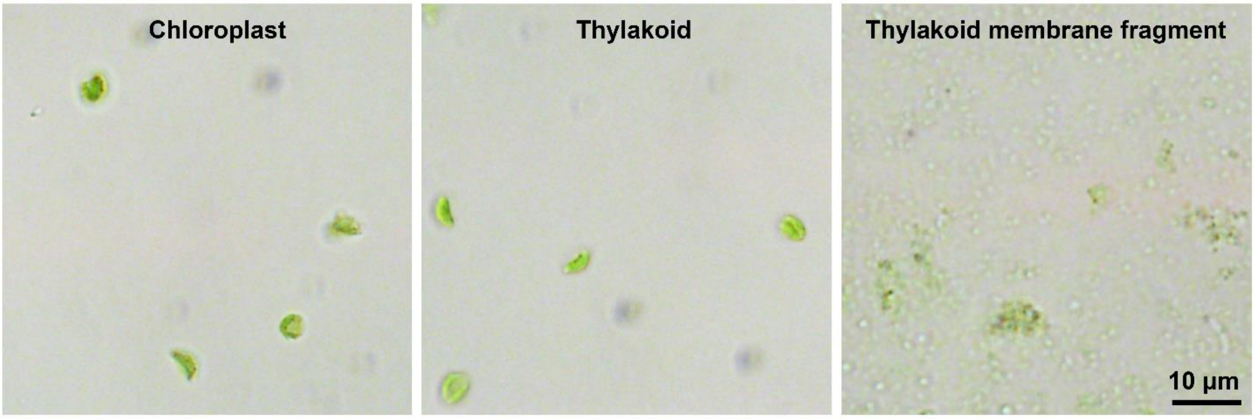

Figure S2. Microscopy images of the chloroplasts, thylakoids, and thylakoid membrane fragments.

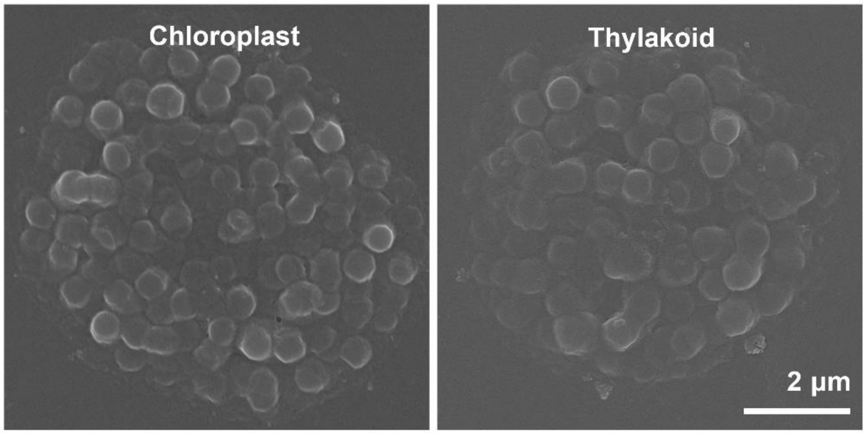

Figure S3. SEM images of the chloroplast and thylakoid.

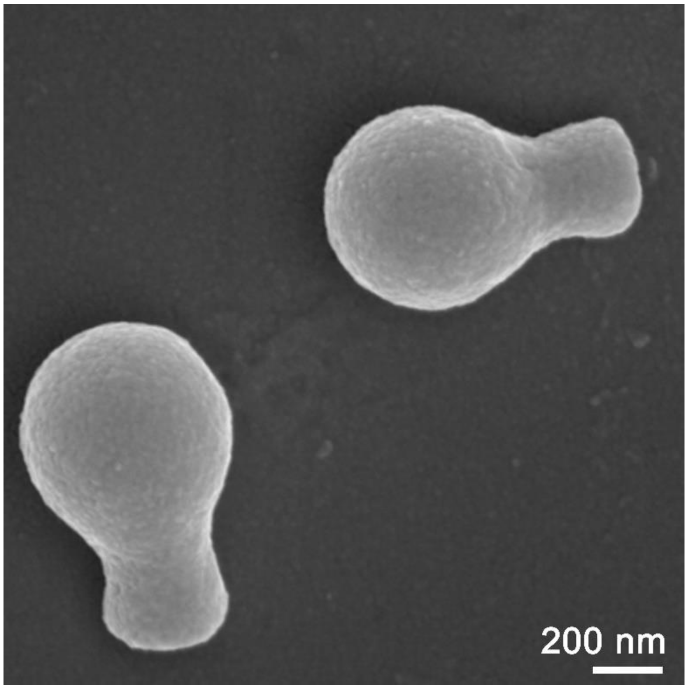

Figure S4. SEM images of the flasklike colloidal particles.

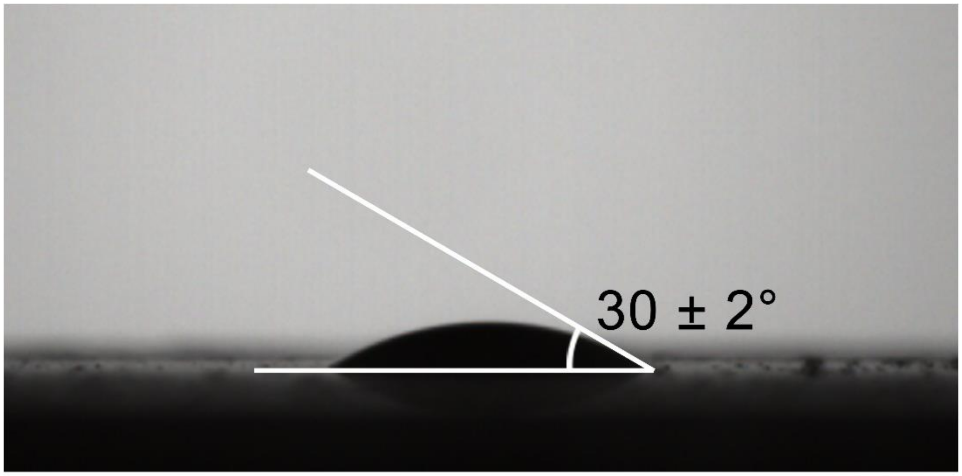

Figure S5. The water contact angle measurement of the flasklike colloidal particle monolayer (30 ± 2°).

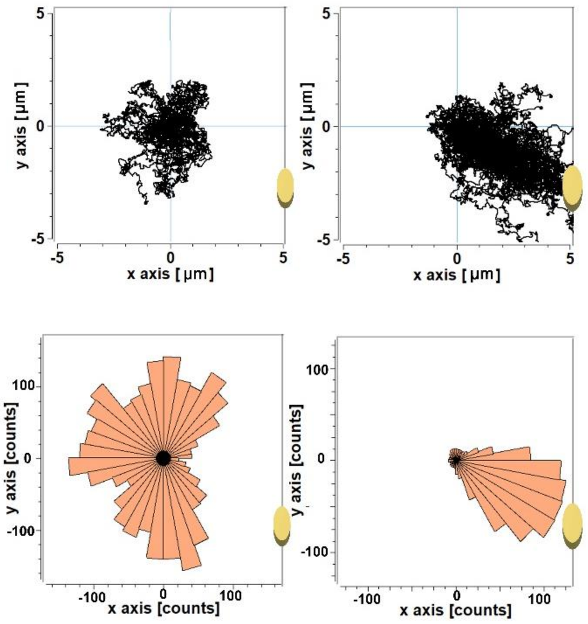

Figure S6. The normalized trajectories of 10 flasklike colloidal particles and flasklike colloidal motors in response to ADP gradient and corresponding angle distribution in 5 s.

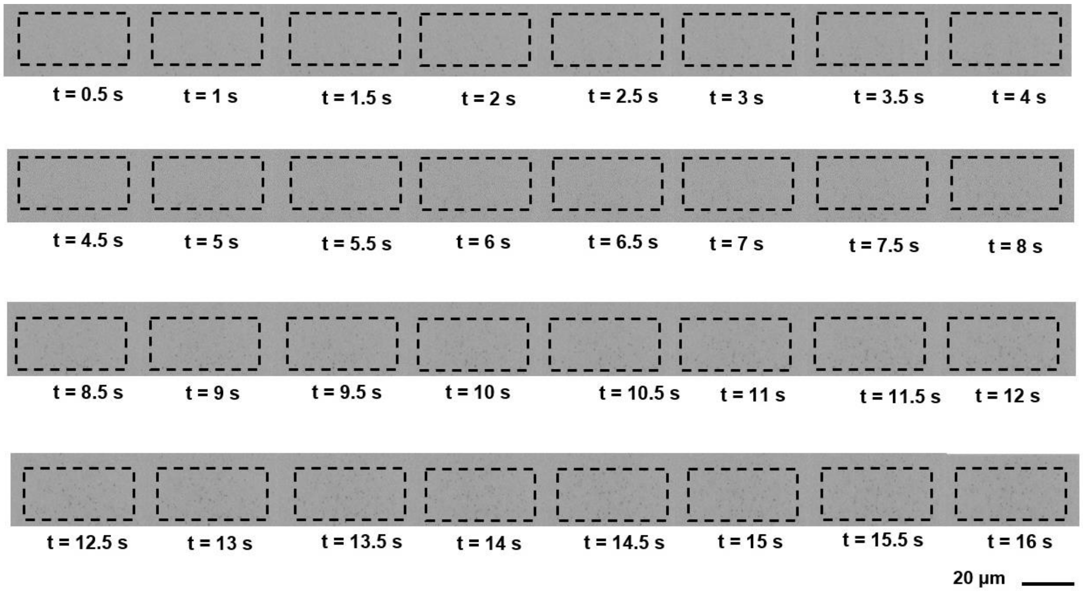

Figure S7. Time-lapse images of the chemotactic process of flasklike colloidal motors in a fixed region.

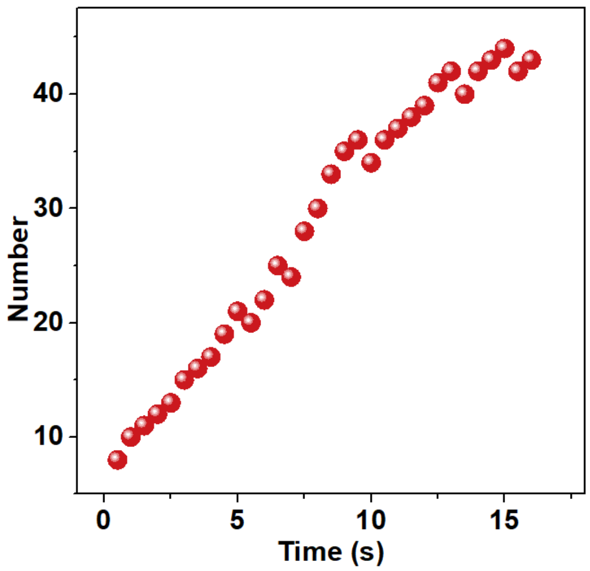

Figure S8. Number of flasklike colloidal motors in the fixed area during chemotaxis in ADP buffer.

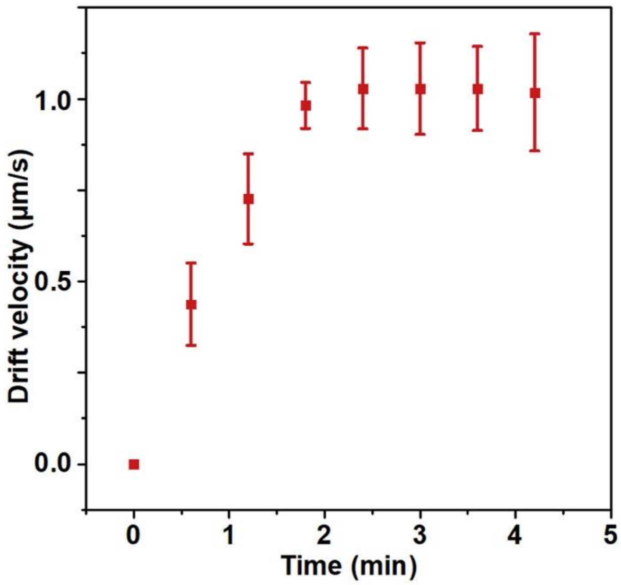

Figure S9. The drift velocity of the flasklike colloidal motor in the microfluidic channel is filled up with ADP buffer.

Supplementary Tables

Table S1. Table Caption of ADP buffer and Acidic buffer component.

Acidic buffer pH = 4.8	Acidic buffer pH = 5.5	Acidic buffer pH = 6.0	Acidic buffer pH = 6.5	Neutral buffer pH = 7.0
20 mM sodium succinate, 5 mM NaH_2PO_4, 2.5 mM $MgCl_2$, 0.6 mM KOH and 5 mM DTT. NaOH titration.	20 mM sodium succinate, 5 mM NaH_2PO_4, 2.5 mM $MgCl_2$, 0.6 mM KOH and 5 mM DTT. NaOH titration.	20 mM sodium succinate, 5 mM NaH_2PO_4, 2.5 mM $MgCl_2$, 0.6 mM KOH and 5 mM DTT. NaOH titration.	20 mM sodium succinate, 5 mM NaH_2PO_4, 2.5 mM $MgCl_2$, 0.6 mM KOH and 5 mM DTT. NaOH titration.	20 mM sodium succinate, 5 mM NaH_2PO_4, 2.5 mM $MgCl_2$, 0.6 mM KOH and 5 mM DTT. NaOH titration.
**ADP buffer pH = 7.0**	**ADP buffer pH = 7.5**	**ADP buffer pH = 8.0**	**ADP buffer pH = 8.5**	**ADP buffer pH = 8.8**
pH 8.0, 10 mM Tricine-NaOH, 20 μM ADP, 5 mM NaH_2PO_4, 2.5 mM $MgCl_2$, and 30 mM NaCl, 5 mM DTT, HCl titration.	pH 8.0, 10 mM Tricine-NaOH, 20 μM ADP, 5 mM NaH_2PO_4, 2.5 mM $MgCl_2$, and 30 mM NaCl, 5 mM DTT, HCl titration.	pH 8.0, 10 mM Tricine-NaOH, 20 μM ADP, 5 mM NaH_2PO_4, 2.5 mM $MgCl_2$, and 30 mM NaCl, 5 mM DTT, NaOH titration.	pH 8.0, 10 mM Tricine-NaOH, 20 μM ADP, 5 mM NaH_2PO_4, 2.5 mM $MgCl_2$, and 30 mM NaCl, 5 mM DTT, NaOH titration.	pH 8.0, 10 mM Tricine-NaOH, 20 μM ADP, 5 mM NaH_2PO_4, 2.5 mM $MgCl_2$, and 30 mM NaCl, 5 mM DTT, NaOH titration.

Supplementary movies S1 to S3

- Motion behavior of flasklike colloidal motors during ATP synthesis at ΔpH = 4 for 5 s. (**Movie S1**)
- Brownian motion behavior of flasklike colloidal motors for 5 s. **(Movie S2**)
- Positive chemotaxis of the flasklike colloidal motors in 60 s(6x) in response to ATP gradient. (**Movie S3**)
